# The Half-Yearly Abstract of the Medical Sciences

**Published:** 1845-11

**Authors:** 


*The Half-Yearly Abstract of the Medical Sciences.* Edited by W. H. RANKING, M. D., Physician to the Suffolk General Hospital. New York: J. & H. G. Langley, 1845.

We have been favored with the first half-yearly number of Vol. I, of this new and valuable periodical. It professes to be "a practical and analytical digest of the contents of the principal British and continental medical works, published in the preceding six months; together with a series of critical reports on the progress of medicine and the collateral sciences, during the same period." In the present number, these professions are excellently well carried out. The half-yearly number contains 372 pages, and presents a complete synopsis of the advances made in the medical sciences for the last six months. Not the least among the many excellencies of the work, is the admirable arrangement of the various excerpta and reports; the convenience of reference being thus materially facilitated. Departments of medical science, but too much neglected in other retrospects, receive in this the notice their growing importance deserves. Among these may be mentioned Pathological Chemistry, Forensic Medicine, Physiology and Microscopic Anatomy, the reports on which, in addition to those of the branches more usually discussed in similar periodicals, are full and satisfactory. Republished at the remarkably low price of $1.00 a year, in advance, we have no hesitation in pronouncing it the cheapest periodical within our knowledge. Subscriptions, &c., to be addressed to the care of J. & H. G. Langley, No. 8, Astor House, New York. Ed.

